# Association of Addition of Ablative Therapy Following Transarterial Chemoembolization With Survival Rates in Patients With Hepatocellular Carcinoma

**DOI:** 10.1001/jamanetworkopen.2020.23942

**Published:** 2020-11-05

**Authors:** Keara English, N. Patrik Brodin, Viswanathan Shankar, Shaoyu Zhu, Nitin Ohri, Yosef S. Golowa, Jacob Cynamon, Sarah Bellemare, Andreas Kaubisch, Milan Kinkhabwala, Shalom Kalnicki, Madhur K. Garg, Chandan Guha, Rafi Kabarriti

**Affiliations:** 1 Department of Radiation Oncology, Montefiore Medical Center and Albert Einstein College of Medicine, Bronx, New York; 2 Department of Radiology, Montefiore Medical Center and Albert Einstein College of Medicine, Bronx, New York; 3 Department of Oncology, Montefiore Medical Center and Albert Einstein College of Medicine, Bronx, New York; 4 Department of Surgery, Montefiore Medical Center and Albert Einstein College of Medicine, Bronx, New York

## Abstract

**Importance:**

Hepatocellular carcinoma (HCC) is a heterogeneous disease with many available treatment modalities. Transarterial chemoembolization (TACE) is a valuable treatment modality for HCC lesions. This article seeks to evaluate the utility of additional ablative therapy in the management of patients with HCC who received an initial TACE procedure.

**Objective:**

To compare the overall survival (OS) and freedom from local progression (FFLP) outcomes after TACE alone with TACE that is followed by an ablative treatment regimen using stereotactic body radiation therapy, radiofrequency ablation, or microwave ablation for patients with HCC.

**Design, Setting, and Participants:**

This cohort study of 289 adults at a single urban medical center examined survival outcomes for patients with nonmetastatic, unresectable HCC who received ablative therapies following TACE or TACE alone from January 2010 through December 2018. The Lee, Wei, Amato common baseline hazard model was applied for within-patient correlation with robust variance and Cox regression analysis was used to assess the association between treatment group (TACE vs TACE and ablative therapy) and failure time events (FFLP per individual lesion and OS per patient), respectively. In both analyses, the treatment indication was modeled as a time-varying covariate. Landmark analysis was used as a further sensitivity test for bias by treatment indication.

**Exposures:**

TACE alone vs TACE followed by ablative therapy.

**Main Outcomes and Measures:**

Freedom from local progression and overall survival. Hypotheses were generated before data collection.

**Results:**

Of the 289 patients identified, 176 (60.9%) received TACE only and 113 (39.1%) received TACE plus ablative therapy. Ablative therapy included 45 patients receiving stereotactic body radiation therapy, 39 receiving microwave ablation, 20 receiving radiofrequency ablation, and 9 receiving a combination of these following TACE. With a median (interquartile range) follow-up of 17.4 (9.5-29.5) months, 242 of 512 (47.3%) lesions progressed, 211 in the group with TACE alone and 31 in the group with TACE plus ablative therapy (*P* < .001). Over 3 years, FFLP was 28.1% for TACE alone vs 67.4% for TACE with ablative therapy (*P* < .001). The 1-year and 3-year OS was 87.5% and 47.1% for patients with lesions treated with TACE alone vs 98.7% and 85.3% for patients where any lesion received TACE plus ablative therapy, respectively (*P* = .01), and this benefit remained robust on landmark analyses at 6 and 12 months. The addition of ablative therapy was independently associated with OS on multivariable analysis for all patients (hazard ratio, 0.26; 95% CI, 0.13-0.49; *P* < .001) and for patients with Barcelona clinic liver cancer stage B or C disease (hazard ratio, 0.31; 95% CI, 0.14-0.69; *P* = .004).

**Conclusions and Relevance:**

Adding ablative therapy following TACE improved FFLP and OS among patients with hepatocellular carcinoma. This study aims to guide the treatment paradigm for HCC patients until results from randomized clinical trials become available.

## Key Points

**Question:**

Is the addition of ablative treatment using stereotactic body radiation therapy, radiofrequency ablation, or microwave ablation to transarterial chemoembolization associated with improved treatment outcomes and survival for patients with hepatocellular carcinoma (HCC)?

**Findings:**

In this cohort study of 289 patients with HCC, the addition of ablative therapy was associated with improved freedom from local progression and overall survival in both univariable and multivariable analyses.

**Meaning:**

These findings suggest that ablative therapy should be considered for patients with hepatocellular carcinoma who have undergone transarterial chemoembolization.

## Teaser

This cohort study examined the association of adding ablative therapy to transarterial chemoembolization with overall survival and freedom from local progression among patients with hepatocellular carcinoma (HCC).

## Introduction

Hepatocellular carcinoma (HCC) is the most common primary liver cancer and is the fourth leading cause of cancer-related death worldwide, accounting for more than 782 000 total deaths in 2018.^1^ Transarterial chemoembolization (TACE) has been shown to improve the survival of patients with HCC compared with best supportive care in randomized clinical trials and meta-analyses conducted over the last 20 years. For example, a 2002 randomized trial^2^ found a survival advantage of conventional TACE when added to best supportive care (TACE, 28.6 months vs best supportive care, 17.9 months; *P* = .01). However, TACE as a solitary treatment rarely produces a complete response. Current consensus guidelines recommend TACE or systemic therapy for patients with either poor performance status, advanced liver cancer, or poor liver function.^3^ The addition of radio frequency ablation (RFA) to TACE likely improves survival in subsets of patients with HCC and should be considered as part of the treatment paradigm for these patients.^4,5,6,7,8^ Whether the addition of other ablative therapies to TACE such as microwave ablation (MWA) or stereotactic body radiation therapy (SBRT) can improve outcomes, including overall survival (OS), is not fully elucidated. Previously published data suggest that SBRT may be associated with a progression-free survival benefit, particularly among patients with a lesser disease burden.^9^

Similarly, MWA may also provide a progression-free survival benefit for subgroups of patients with HCC when combined with TACE based on previous reports.^10,11^ In this study, we evaluate the association of local ablative therapy using SBRT, MWA, or RFA given after TACE with the overall survival benefit. Additionally, this study investigates the association of local therapy with changes in individual HCC lesions by examining freedom from local progression (FFLP).

## Methods

### Patient Selection

In this retrospective cohort study approved by the Albert Einstein College of Medicine institutional review board, we utilized an in-house developed software application to identify all patients age 18 years or older diagnosed with HCC from a single urban medical center from January 1, 2010, to December 31, 2018, who received TACE as their initial treatment. The data were analyzed between February and June 2020. Based on these inclusion criteria, patients who were treated with upfront systemic therapy, surgery, transplantation, or ablation without prior TACE were not included in this study. Diagnosis was based on histology or imaging criteria diagnostic of HCC, based on Liver Reporting & Data System (LI-RADS) criteria. At our institution, all patients with HCC are presented in a multidisciplinary tumor board, and treatment management is decided after discussion among boardmembers from different specialties. The study was exempted from informed consent requirements because it was a retrospective medical record review of existing data among patients already treated at our institution. Additionally, patients were excluded if they did not receive follow-up imaging after the initial TACE procedure. This study followed the Strengthening the Reporting of Observational Studies in Epidemiology (STROBE) reporting guideline for cohort studies.

### Data Collection

The following information was obtained for each patient: demographic characteristics, tumor size and location, number of lesions, presence of tumor thrombus, use of systemic therapy, laboratory measures, Eastern Cooperative Oncology Group (ECOG) performance status, Model for End-stage Liver Disease (MELD), socioeconomic status (derived from factors such as median household income and level of education in a specific area code, with a lower score reflecting lower socioeconomic status), Child-Turcotte-Pugh scores,^12^ and treatment dates and modality for each lesion within a patient.

FFLP was defined as the absence of local disease progression by Response Evaluation Criteria in Solid Tumors (RECIST) criteria on follow-up imaging.^13^ The treating physician determined the frequency of follow-up imaging but typically follows recommended guidelines for obtaining imaging at least every 3 months for the first year and at extending intervals thereafter based on the clinical scenario. The event time in the FFLP analysis was censored at the time of recurrence, last follow-up, death, or at the time of liver transplantation, whichever came first. For the OS analysis, failure time was defined as the interval between the time of first TACE treatment and death or censoring at the time of the last contact. The patients were categorized as being treated with TACE plus ablative therapy if any lesion received SBRT, RFA, or MWA. Treatments involving selective internal radiation therapy (eg, with yttrium-90) were not included in this analysis.

### Statistical Analysis

Distribution of patients’ demographic and clinical characteristics was summarized using descriptive statistics by treatment groups (TACE alone vs TACE plus ablation). The variable representing patients who received ablation therapy has a varying initiation time, which was handled as a time-dependent covariate in both lesion level (FFLP) and patient-level (OS) analysis. Those who received ablation were considered in the TACE alone group until the time of ablation initiation and then in the TACE plus ablation group from time of initiation on. The time from first TACE treatment to FFLP and time to death from the first TACE date was computed using a Simon and Makuch plot,^14^ and the survival probabilities were compared using a Mantel-Byar test^15^ for the time-dependent treatment group variable. For the other time-fixed covariates, the survival probabilities were estimated using the Kaplan-Meier method along with log-rank statistics. Because lesions are clustered within patients, a score (sandwich) test was used to compare the survival distributions.

In the FFLP analysis, failure times come from 1 or more lesions within a patient, thus producing correlated failure times. The Lee, Wei, Amato common baseline marginal hazard model^16^ with an independent working correlation structure was fitted to evaluate the association of treatment group with FFLP. A robust sandwich variance was used to adjust for within-patient correlation. Stratified analysis was performed comparing how the addition of ablation affected the FFLP of lesions when stratified by maximum tumor diameter 2 cm or less vs greater than 2 cm.

A Cox regression model with time-independent and time-dependent covariates was used to evaluate the association of treatment group with OS. Potential confounders that were associated with the outcome on univariate models at a 2-sided *P* < .25 were considered for the multivariable models. The proportional hazards assumptions were examined via visual inspection and a formal test of the Schoenfeld residuals. A subgroup analysis was performed comparing the OS between patients receiving TACE alone vs TACE plus ablative therapy for those with Barcelona clinic liver cancer (BCLC) stage B or C disease.

Approximately 22% of the patients (recorded in terms of individual lesions) had incomplete information for some covariate, which can produce biased or inefficient results under complete case analysis. We used a multiple imputation approach to handle missing data under a missing at random assumption. We implemented a flexible, fully conditional specification imputation approach to accommodate different variable scales and random missingness using 20 data sets. We adapted the White and Royston method^17^ for the imputation model by including all covariates, failure statuses, and the Nelson-Aalen marginal cumulative hazard as predictors. Sensitivity analyses were performed in the form of landmark analyses at 6 and 12 months after the first TACE procedure to further account for potential immortal time bias between the patients receiving TACE alone vs TACE plus ablative therapy. All statistical analyses were performed using SAS version 9.4 (SAS Institute Inc) or STATA version 14 (StataCorp). Statistical significance was determined at 2-sided α = .05.

## Results

### Patient Demographic Characteristics

Two hundred eighty-nine patients were identified from 2010 to 2018, with a total of 512 lesions that met inclusion criteria (Table 1). The distribution of patient and clinical characteristics are presented in Table 1. The mean (SD) age was 63.6 (8.1) years, and the majority were men (207 patients [71.6%]). Of all 289 patients, 133 (46.0%) identified as Hispanic, 70 (24.2%) as non-Hispanic Black, 31 (10.7%) as non-Hispanic White, and 55 (19%) and as non-Hispanic other. The median (interquartile range [IQR]) socioeconomic score in the cohort was −3.15 (IQR −5.97 to −1.00).

**Table 1.** Patient and Tumor Characteristics in the Overall Cohort

Baseline characteristics	Patients, No. (%)		*P* value
	TACE alone (n = 176)	TACE plus ablative therapy (n = 113)	
Age, mean (SD), y	63.4 (8.5)	63.8 (7.6)	.68
Age group, y			
<65	105 (59.7)	61 (53.9)	.34
≥65	71 (40.3)	52 (46.0)	
Sex			
Male	130 (73.9)	77 (68.1)	.29
Female	46 (26.1)	36 (31.9)	
Race/ethnicity			
Hispanic	79 (44.8)	54 (47.8)	.47
Non-Hispanic White	17 (9.7)	14 (12.4)	
Non-Hispanic Black	48 (27.3)	22 (19.5)	
Non-Hispanic Other^a^	32 (18.2)	23 (20.4)	
ECOG performance status			
0	64 (36.4)	48 (42.5)	.70
1	71 (40.3)	43 (38.1)	
≥2	25 (14.2)	15 (13.3)	
Missing (not included in χ^2^-test)	16 (9.1)	7 (6.2)	
Socioeconomic status, median (IQR)^b^	−2.64 (−6.21 to −1.05)	−2.37 (−5.79 to −0.98)	.62
Child-Turcotte-Pugh category			
A, mild	95 (54.0)	76 (67.3)	.01
B	67 (38.1)	35 (30.9)	
C, severe	14 (7.9)	1 (0.9)	
Missing (not included in χ^2^-test)	0	1 (0.9)	
α-Fetoprotein at diagnosis, ng/mL			
<10	63 (35.8)	46 (40.7)	.47
≥10	102 (57.9)	62 (54.9)	
Missing (not included in χ^2^-test)	11 (6.3)	5 (4.4)	
MELD score at diagnosis, median (IQR)^c^	10.9 (8.2 to 14.3)	9.9 (7.5 to 12.4)	.08
Tumor thrombus			
No	117 (66.5)	66 (58.4)	.42
Yes	47 (26.7)	33 (29.2)	
Missing (not included in χ^2^-test)	12 (6.8)	14 (12.4)	
Lesions, No.			
1	68 (38.6)	42 (37.2)	.47
2-3	65 (36.9)	49 (43.4)	
>3	43 (24.4)	22 (19.5)	
Maximum tumor diameter, median (IQR), cm	2.8 (2.0-4.2)	2.4 (2.0-3.3)	.10
Tumors, No. (%)			
≤2	49 (27.8)	43 (38.1)	.08
>2	125 (71.0)	70 (61.9)	
Missing (not included in χ^2^ test)	2 (1.2)	0	

Abbreviations: ECOG, Eastern Cooperative Oncology Group; IQR, interquartile range; MELD, Model for End-stage Liver Disease; TACE, transarterial chemoembolization.

SI conversion factor: To convert α-Fetoprotein to μg/L, multiply by 1.0.

^a^ Race classifications included in this category included Asian, Native Hawaiian or other Pacific Islander, or other.

^b^ Score derived from factors such as median household income and level of education in a specific area code, with a lower score reflecting lower socioeconomic status.

^c^ MELD score assesses liver disease severity and candidacy for transplant.

Among patients treated with TACE alone, 95 (54.0%), 67 (38.1%), and 14 (7.9%) were classified with Child-Turcotte-Pugh Class A, Class B, and Class C, respectively. This differed from the TACE plus ablative therapy group, which was composed of 76 (67.8%), 36 (31.3%), and 1 (0.9%) patients in classes A, B, and C, respectively. The median (IQR) MELD score did not significantly differ between groups. It was 10.9 (8.2-14.3) in the TACE alone group and 9.9 (7.5-12.4) in the TACE plus ablative therapy group. Most patients had an ECOG performance status of either 0 (112 patients [42.1%]) or 1 (114 patients [42.9%]), with 40 patients (15.0%) categorized as having an ECOG status of 2 or greater, and this was not significantly different between the 2 treatment groups. The tumor burden per patient, categorized as either 1, 2 to 3, or greater than 3 lesions, did not differ between the treatment groups (Table 1).

### TACE vs TACE Plus Ablative Therapy

Patients in this cohort had a median of 3 lesions (range, 1-10), but the median number of lesions with TACE therapy considered for this analysis was 2 (range, 1-8). Of the 289 patients, 176 patients received TACE, and 113 (40.4%) patients received TACE plus ablative therapy. Classifying this group by specific therapy, 45 (39.8%) patients received SBRT, 39 (34.5%) received microwave ablation, and 20 (17.7%) RFA received as their primary ablation, while 9 (7.9%) received combination ablation therapy following TACE. Of patients receiving ablative therapy, 94 received ablative therapy to only 1 lesion, and 19 received ablative therapy to 2 or more lesions. The median number of TACE procedures per lesion was 1 (range, 1-5) and the median number of TACE procedures per patient was 2 (range, 1-13). Median (IQR) time from diagnosis to first TACE was 1.5 (0.8-2.1) months, and the median time from diagnosis to first ablative therapy was 5.5 (3.7-10.2) months. The median (IQR) time to ablative therapy after the first TACE was 3.5 (2.1-8.1) months. The median (IQR) frequency of follow-up imaging post-TACE was 2 (1-5) visits, and the median imaging frequency postablation was 1 (1-6) visit. The median (IQR) time interval between TACE and first, second, and third imaging was 1.4 (1.1-2.6) months, 1.1 (1.1-2.3) months, and 1.4 (1.2-2.2) months, respectively. Similarly, the interval between postablation therapy and first, second, and third imaging was 1.8 (1.4-2.5) months, 1.8 (1.4-2.5) months, and 1.8 (1.5-2.5) months, respectively. For the entire cohort, the median follow-up time was 17.4 (IQR, 9.6-29.8; range, 0.07-87.3) months per individual. A total of 36 patients received a liver transplant, 25 of 176 (14.2%) in the group of patients receiving TACE alone and 11 of 113 (9.7%) in the group receiving TACE plus ablative therapy.

### Freedom From Local Progression

**Figure 1.**
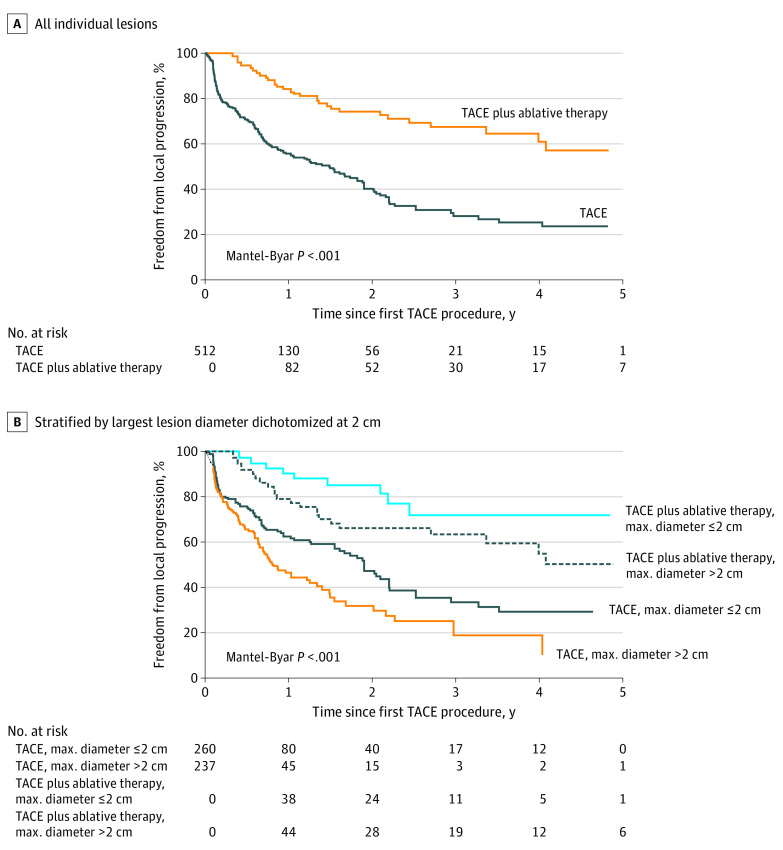
Comparison of Freedom From Local Progression (FFLP) Between Transarterial Chemoembolization (TACE) Alone and TACE Plus Ablative Therapy

The median time to progression was 20.8 (95% CI, 16.1-25.2) months, and of the 512 lesions, 242 (47.3%) had progressed on follow-up imaging, including 211 of 379 (55.7%) lesions in the group with TACE alone and 31 of 133 (23.3%) in the group with TACE plus ablative therapy (*P* < .001) (Figure 1). The proportion of lesions without local progression (FFLP) at 1, 2, 3, and 5 years were 59.2% (95% CI, 54.4%-63.7%), 46.7% (95% CI, 41.4%-51.8%), 37.4% (95% CI, 31.7%-43.1%), and 31.6% (95% CI, 25.2%-38.1%), respectively. The proportion of lesions receiving TACE alone without local progression was 55.7% (95% CI, 51.9%-62.4%), 40.1% (95% CI, 33.7%-46.4%), 28.1% (95% CI, 21.2%-35.4%), and 23.6% (95% CI, 16.4%-31.5%) at 1, 2, 3, and 5 years respectively, whereas this was 84.2% (95% CI, 74.7%-90.3%), 74.2% (95% CI, 63.7%-82.0%), 67.4% (95% CI, 55.8%-76.6%), and 57.1% (95% CI, 42.0%-69.6%) for those receiving TACE plus ablative therapy (*P* < .001 for the Mantel-Byar test). When patients were stratified by lesion size 2 cm and less or greater than 2 cm, the addition of ablative therapy to TACE resulted in a significant improvement in FFLP for both size categories (eg, FFLP at 1 year with lesion size >2 cm: TACE with ablative therapy, 79.0%; 95% CI, 64.4%-88.2% vs TACE only, 46.5%; 95% CI, 37.9%-54.6%; *P* < .001) (Figure 1). This result remained the same when stratifying using a 3 cm cutoff. Furthermore, a subgroup analysis showed that the addition of ablative therapy was also associated with significantly improved FFLP for lesions that appeared to have a complete response on imaging after the first TACE procedure (eg, FFLP at 3 years: TACE with ablative therapy, 85.3%; 95% CI, 60.4%-95.1% vs TACE only, 45.7%; 95% CI, 31.1%-59.1%; *P* = .02) (eFigure 1 in the Supplement). On univariable analysis for FFLP, the hazard ratio (HR) for TACE plus ablative therapy vs TACE alone was 0.39 (95% CI, 0.25-0.62; *P* < .001) (eTable 1 in the Supplement). On multivariable analysis, the addition of ablative therapy was associated with better FFLP (HR, 0.34; 95% CI, 0.21-0.53; *P* < .001) (Table 2) as compared with TACE alone. Age, sex, race/ethnicity, Child-Turcotte-Pugh category, and maximum tumor diameter were also found to be independent predictors of FFLP (Table 2).

**Table 2.** Multivariable Complete Case Analysis and Multiple Imputation Analysis Estimates for Freedom From Local Progression From Common Baseline Hazard Model

Variable	Complete case estimates (n = 512 lesions)		Multiple imputation estimates (n = 512 lesions)^a^	
	Hazard ratio (95% CIs)	*P* value	Hazard ratio (95% CIs)	*P* value
Treatment group (time-dependent covariate)				
TACE alone	1 [Reference]	<.001	1 [Reference]	<.001
TACE plus ablative therapy	0.33 (0.21-0.52)		0.34 (0.21-0.53)	
Age, y				
<65	1 [Reference]	.03	1 [Reference]	.01
≥65	1.49 (1.04-2.12)		1.57 (1.11-2.22)	
Sex				
Men	1 [Reference]	.02	1 [Reference]	.08
Women	0.56 (0.35-0.91)		0.66 (0.41-1.05)	
Race/ethnicity				
Non-Hispanic White	1 [Reference]		1 [Reference]	
Hispanic	0.57 (0.36-0.90)	.02	0.58 (0.36-0.91)	.02
Non-Hispanic Black	0.76 (0.47-1.23)	.26	0.85 (0.52-1.40)	.53
Non-Hispanic other^b^	0.56 (0.31-0.99)	.048	0.60 (0.34-1.06)	.08
Child-Turcotte-Pugh category				
A	1 [Reference]		1 [Reference]	
B	1.04 (0.69-1.57)	.84	1.06 (0.70-1.60)	.80
C	2.40 (1.23-4.65)	.01	2.46 (1.26-4.82)	.01
MELD score per 1 unit increase	1.03 (0.98-1.07)	.26	1.03 (0.99-1.07)	.17
Maximum tumor diameter, cm				
≤2	1 [Reference]	.01	1 [Reference]	.01
>2	1.50 (1.11-2.03)		1.47 (1.10-1.95)	

Abbreviations: MELD, Model for End-stage Liver Disease; TACE, transarterial chemoembolization.

^a^ Estimates performed based on multiple imputation using 20 data sets.

^b^ Race classifications included in this category included Asian, Native Hawaiian or other Pacific Islander, or other.

### Overall Survival

**Figure 2.**
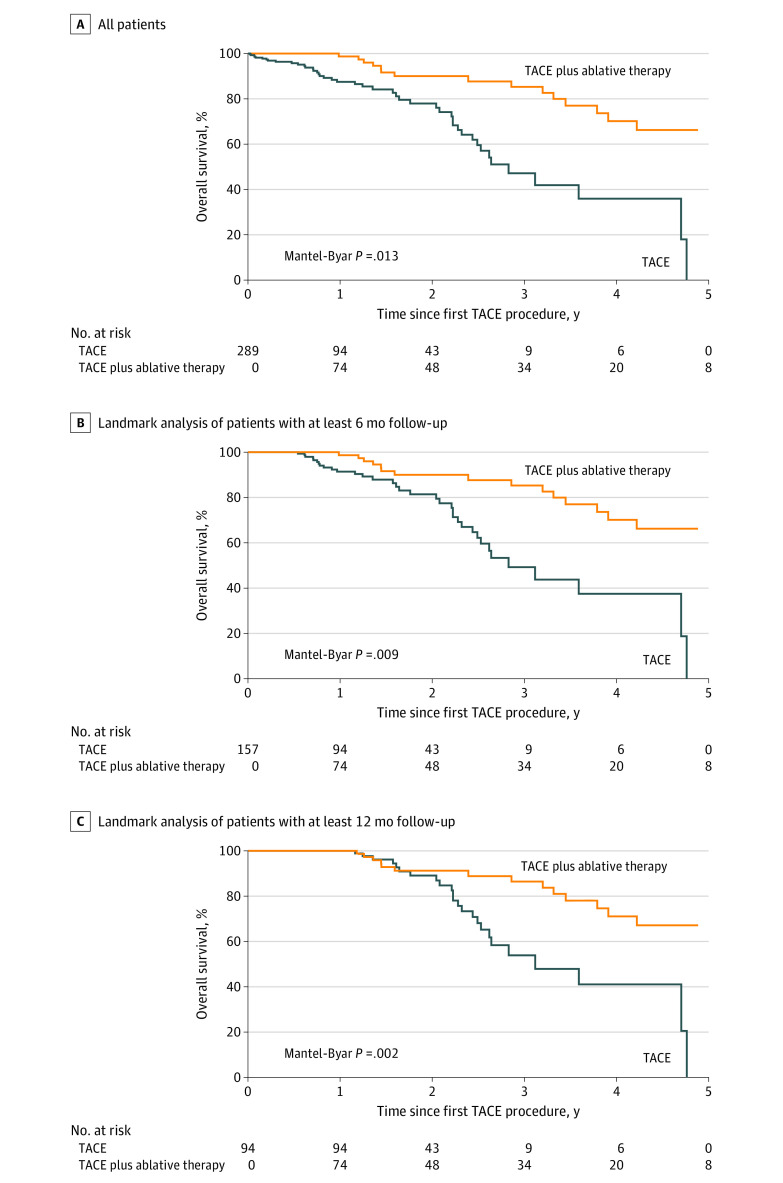
Comparison of Overall Survival (OS) Between Transarterial Chemoembolization (TACE) Alone and TACE Plus Ablative Therapy

Out of 289 patients, 61 (21.1%) died within the study time frame, and the median (SE) survival was 56.4 (6.3) months. The median (SE) survival following initial TACE procedure was 33.9 (3.1) months, and the median (SE) survival after TACE plus ablative therapy was 83.4 (8.0) months. The OS among patients receiving TACE alone was 87.5% (95% CI, 81.1%-91.8%), 77.9% (95% CI, 68.3%-85.0%), and 47.1% (95% CI, 32.0%-60.9%), and for patients with TACE plus ablative therapy was 98.7% (95% CI, 91.0%-99.8%), 90.0% (95% CI, 80.2%-95.1%), and 85.3% (95% CI, 73.0%-92.3%) at 1, 2, and 3 years, respectively (*P* = .01 for the Mantel-Byar test) (Figure 2). Additionally, the Kaplan-Meier survival curves using landmark analyses only including those patients with at least 6 or 12 months follow-up, respectively, also showed improved OS for patients receiving ablative therapy (Figure 2).

On univariable analysis, the HR for TACE plus ablative therapy vs TACE alone was 0.28 (95% CI, 0.15-0.52; *P* < .001) (eTable 2 in the Supplement). The addition of ablative therapy was associated with OS on multivariable analysis (HR, 0.26; 95% CI, 0.13-0.49; *P* < .001) (Table 3). Furthermore, Child-Turcotte-Pugh category (Class B vs Class A: HR, 1.93; 95% CI, 1.12-3.32; *P* = .30) and AFP (HR, 1.84; 95% CI, 1.01-3.37; *P* = .048) were also associated with OS (Table 3).

**Table 3.** Multivariable Complete Case Analysis and Multiple Imputation Analysis Estimates for Overall Survival From Cox Regression Model

Variable	Complete case estimates (n = 289 lesions)		Multiple imputation estimates (n = 289 lesions)^a^	
	Hazard ratio (robust 95% CIs)	*P* value	Hazard ratio (robust 95% CIs)	*P* value
Treatment group (time dependent covariate)				
TACE alone	1 [Reference]	<.001	1 [Reference]	<.001
TACE plus ablative therapy	0.26 (0.13-0.52)		0.26 (0.13-0.49)	
Age, y				
<65	1 [Reference]	.05	1 [Reference]	.06
≥65	1.75 (1.00-3.08)		1.69 (0.98-2.89)	
Sex				
Men	1 [Reference]	.60	1 [Reference]	.77
Women	1.20 (0.62-2.31)		1.10 (0.58-2.08)	
Race/ethnicity				
Non-Hispanic White	1 [Reference]		1 [Reference]	
Hispanic	0.65 (0.24-1.81)	.41	0.66 (0.25-1.79)	.42
Non-Hispanic Black	1.27 (0.43-3.72)	.66	1.37 (0.48-3.89)	.56
Non-Hispanic other^b^	1.04 (0.32-3.35)	.94	0.97 (0.31-3.10)	.96
Child-Turcotte-Pugh category				
A	1 [Reference]		1 [Reference]	
B	1.84 (1.05-3.22)	.03	1.93 (1.12-3.32)	.02
C	2.52 (0.55-11.6)	.23	2.24 (0.49-10.2)	.30
α-Fetoprotein at diagnosis, ng/mL				
<10	1 [Reference]	.03	1 [Reference]	.048
≥10	1.95 (1.07-3.56)		1.84 (1.01-3.37)	
Socioeconomic status per 1 unit increase	1.09 (0.99-1.20)	.08	1.07 (0.97-1.17)	.16

Abbreviation: TACE, transarterial chemoembolization.

SI conversion factor: To convert α-Fetoprotein to μg/L, multiply by 1.0.

^a^ Data include 20 cases with multiple imputation.

^b^ Race classifications included in this category included Asian, Native Hawaiian or other Pacific Islander, or other.

In a subgroup analysis, the addition of ablative therapy was also an independent predictor of improved OS for patients with BCLC stage B or C, with a multivariable HR of 0.31 (95% CI, 0.14-0.69; *P* = .004) (eTable 3 in the Supplement; and Kaplan-Meier survival curves are shown in eFigure 2 in the Supplement).

## Discussion

We found that the addition of an ablative treatment modality using either SBRT, MWA, or RFA following an initial TACE procedure in patients with newly diagnosed HCC was associated with improved overall survival and freedom from local progression. These results were consistent with multivariable and landmark analyses, indicating a robust association. Furthermore, this benefit persisted in subgroup analyses in patients with BCLC stage B or C and in those with a complete response on imaging following TACE.

The addition of systemic therapies such as sorafenib or brivanib to TACE has been shown not to improve survival in patients with HCC.^18,19^ Randomized data regarding the combination of ablative therapies, especially those using SBRT or MWA, with TACE is limited. The addition of RFA to TACE has been shown to improve outcomes in patients with HCC,^4,5,20,21^ which is consistent with our results. Although RFA is more widely accepted as definitive or salvage therapy, external beam radiotherapy (including SBRT) is an emerging modality for the management of these patients. SBRT appears to result in similar local control rates and overall survival outcomes^22^ with a potential advantage for treating larger tumors with improved local control of larger HCC lesions compared with RFA.^23^ In this study, the benefit of ablative therapy was shown to be consistent for both smaller and larger HCC lesions. As SBRT was the most common ablative modality used in this cohort, this could explain our results of the benefit that persisted even in tumors that were larger than 3 cm.

The role of radiation therapy in the management of HCC continues to emerge with recent data showing improvement in survival when using RT in combination with partial hepatectomy compared with partial hepatectomy alone for patients with portal vein tumor thrombus.^24^ Benefits have also been shown for the addition of TACE and RT with sorafenib compared with sorafenib alone in patients with macroscopic vascular invasion.^25^ Here we show that a similar benefit exists when adding SBRT or other ablative therapies to TACE compared with TACE alone. We are still awaiting the results of RTOG 1112, a trial that randomized patients to sorafenib alone vs SBRT followed by sorafenib, which will further elucidate the utility of SBRT in the treatment paradigm for hepatocellular carcinoma.

The FFLP (1 year, 55.7%; 3 year, 28.1%) and OS rates (1 year, 87.5%; 2 year, 77.9%; and 3 year, 47.1%) among the patients receiving TACE alone in this study are comparable with or greater than the data reported in the literature. For example, the randomized control trial presented by Llovet et al^2^ cited a 2-year OS rate of 63% and a rate of objective responses sustained for at least 6 months of 35%, and another randomized trial by Lo et al^26^ cited a 39% objective response rate and 3-year OS rate of 26% in their TACE-only patient cohort. Given the low rates of local disease control with TACE alone in our and other studies, we show improvement not only in FFLP with adding ablative therapies to TACE but also in survival regardless of the BCLC stage.

A secondary endpoint of our study was to evaluate the utility of ablative therapy following TACE explicitly among patients fitting the BCLC stage B or C criteria. Specifically, we sought to determine if adding ablative therapy following TACE resulted in improved OS among this subgroup of patients. Currently, recommendations for patients in this category consist of TACE or sorafenib per the BCLC guidelines. A notable strength of our study is the large, real-world cohort sample size from a patient population in a large urban medical center with a vast amount of diversity and representative of a population with poor socioeconomic status. Our results indicate that adding ablative therapy following TACE should be considered in the management of patients with BCLC satge B or C disease.

### Limitations

There are some notable limitations to this study. First, the study design was retrospective, which could have introduced selection bias based on unknown factors not identified during the chart review. We used multivariable Cox regression and subgroup analyses to account for this potential selection bias between treatment groups. Second, some data were missing for a small number of patients who declined further treatment, missed appointments and appropriate imaging follow-up, or did not have their functional status documented in their medical records; some demographic information might be missing because patient data were not available in their records. We performed multiple imputation analyses to account for this. There is also a potential for immortal time bias to skew the association with addition of ablative therapy and outcome because of the interval between TACE and subsequent ablative therapy. This was accounted for by handling the treatment group as a time-dependent covariate with patients assigned to TACE alone until the time they received ablative therapy. We furthermore performed landmark analyses at 6 and 12 months after the first TACE procedure in our sensitivity analyses, which showed that there is still a significant survival benefit of adding ablative therapy even when accounting for this effect.

## Conclusions

The present study showed statistically significant improved survival outcomes and freedom from local progression among HCC patients who received ablative therapy in addition to TACE when compared with those who only received TACE without ablative therapy. Therefore, ablative therapy for individual lesions should be considered during HCC treatment planning for patients who can tolerate ablation, regardless of the initial response to TACE. Our findings also suggest additional therapeutic utility of ablative therapies including SBRT for BCLC stage B and C patients.

## 

## Notes

Supplement. **eFigure 1.** A Subgroup Analysis of FFLP Including Only Those Lesions That Had a Complete Imaging Response After the First TACE Procedure **eFigure 2.** A Subgroup Analysis of OS Including Only the Patients With BCLC Stage B or C Disease **eTable 1.** Univariable Complete Case (CC) Analysis and Multiple Imputation (MI) Analysis Estimates for Freedom From Local Progression (FFLP) From LWA Model: Unadjusted Hazard Ratio, Robust 95% CI and P Value **eTable 2.** Univariable Complete Case (CC) Analysis and Multiple Imputation (MI) Analysis Estimates for Overall Survival (OS) From Cox Regression Model: Unadjusted Hazard Ratio, 95% CI and P Value **eTable 3.** Multivariable Complete Case (CC) Analysis and Multiple Imputation (MI) Analysis Estimates for Overall Survival (OS) From Cox Regression Model in Patients With BCLC Stage B or C: Adjusted Hazard Ratio, Robust 95% CI and P Value
