# Supplementary material for: Association of Addition of Ablative Therapy Following Transarterial Chemoembolization With Survival Rates in Patients With Hepatocellular Carcinoma
Source: JAMA Netw Open. 2020 Nov 5;3(11):e2023942. doi: 10.1001/jamanetworkopen.2020.23942 (PMC7645696; doi:10.1001/jamanetworkopen.2020.23942)
Supplement: Supplement. — eFigure 1. A Subgroup Analysis of FFLP Including Only Those Lesions That Had a Complete Imaging Response After the First TACE Procedure eFigure 2. A Subgroup Analysis of OS Including Only the Patients With BCLC Stage B or C Disease eTable 1. Univariable Complete Case (CC) Analysis and Multiple Imputation (MI) Analysis Estimates for Freedom From Local Progression (FFLP) From LWA Model: Unadjusted Hazard Ratio, Robust 95% CI and P Value eTable 2. Univariable Complete Case (CC) Analysis and Multiple Imputation (MI) Analysis Estimates for Overall Survival (OS) From Cox Regression Model: Unadjusted Hazard Ratio, 95% CI and P Value eTable 3. Multivariable Complete Case (CC) Analysis and Multiple Imputation (MI) Analysis Estimates for Overall Survival (OS) From Cox Regression Model in Patients With BCLC Stage B or C: Adjusted Hazard Ratio, Robust 95% CI and P Value [file jamanetwopen-e2023942-s001.pdf]

Supplemental Online Content

English K, Brodin NP, Shankar V, et al. Association of addition of ablative therapy following transarterial chemoembolization with survival rates in patients with hepatocellular carcinoma. *JAMA Netw Open*. 2020;3(11):e2023942. doi:10.1001/jamanetworkopen.2020.23942

eFigure 1. A Subgroup Analysis of FFLP Including Only Those Lesions That Had a Complete Imaging Response After the First TACE Procedure
eFigure 2. A Subgroup Analysis of OS Including Only the Patients With BCLC Stage B or C Disease
eTable 1. Univariable Complete Case (CC) Analysis and Multiple Imputation (MI) Analysis Estimates for Freedom From Local Progression (FFLP) From LWA Model: Unadjusted Hazard Ratio, Robust 95% CI and *P* Value
eTable 2. Univariable Complete Case (CC) Analysis and Multiple Imputation (MI) Analysis Estimates for Overall Survival (OS) From Cox Regression Model: Unadjusted Hazard Ratio, 95% CI and *P* Value
eTable 3. Multivariable Complete Case (CC) Analysis and Multiple Imputation (MI) Analysis Estimates for Overall Survival (OS) From Cox Regression Model in Patients With BCLC Stage B or C: Adjusted Hazard Ratio, Robust 95% CI and *P* Value

This supplemental material has been provided by the authors to give readers additional information about their work.

© 2020 English K et al. *JAMA Network Open.*

eFigure 1. A subgroup analysis of FFLP including only those lesions that had a complete imaging response after the first TACE procedure.

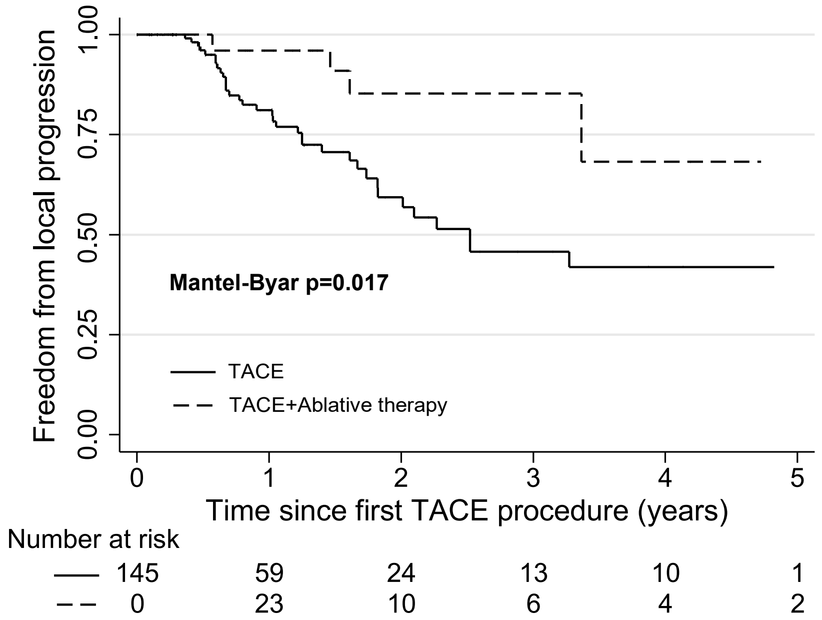

eFigure 2. A subgroup analysis of OS including only the patients with BCLC stage B or C disease.

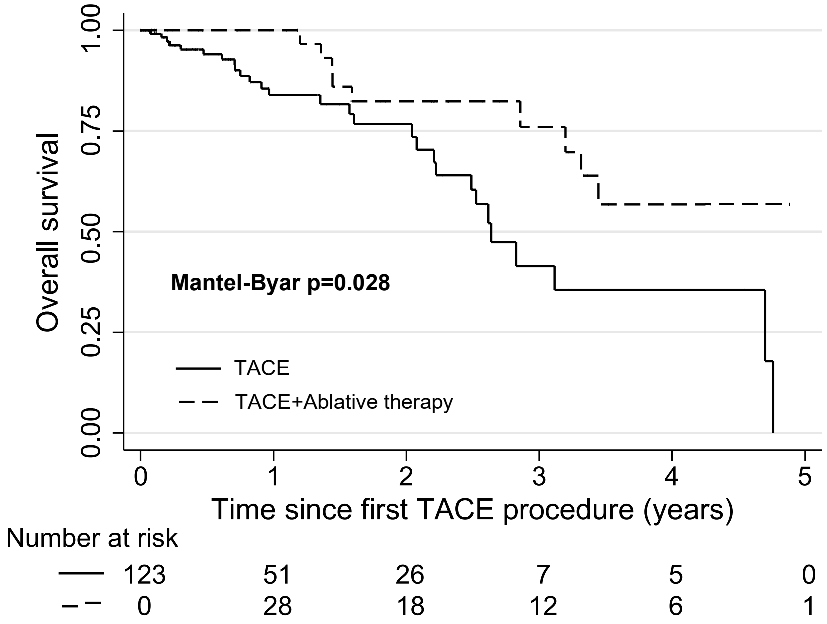

© 2020 English K et al. *JAMA Network Open.*

eTable1. Univariable complete case (CC) analysis and Multiple Imputation (MI) analysis estimates for freedom from local progression (FFLP) from LWA model: unadjusted Hazard Ratio, robust 95% CI and P-value.

	**Complete Case Estimates (*n* = 512 lesions)**		**Multiple Imputation Estimates (*n* = 512 lesions, M=20)**	
**Variable**	**Hazard ratio (robust 95% CIs)**	**P-value**	**Hazard ratio (robust 95% CIs)**	**P-value**
Treatment group (Time Dependent Covariate) (TACE + ablative therapy = 1, TACE alone = 0)	0.39 (0.25 , 0.62)	<0.001	0.39 (0.25 , 0.62)	<0.001
Age group (≥65y = 1, <65y = 0)	1.26 (0.88 , 1.79)	0.21	1.26 (0.0.88 , 1.79)	0.21
Sex (female = 1, male = 0)	0.67 (0.43 , 1.05)	0.080	0.67 (0.43 , 1.05)	0.080
Race/Ethnicity				
Non-Hispanic White	1.0 (ref)		1.0 (ref)	
Hispanics	0.61 (0.38, 0.99)	0.049	0.61 (0.38, 0.99)	0.049
Non-Hispanic Black	0.89 (0.53, 1.48)	0.65	0.89 (0.53, 1.48)	0.65
Non-Hispanic Other	0.59 (0.34, 1.06)	0.077	0.59 (0.34, 1.06)	0.077
ECOG performance status				
0	1.0 (ref)		1.0 (ref)	
1	1.10 (0.75, 1.63)	0.63	1.10 (0.76, 1,61)	0.61
≥2	1.41 (0.86, 2.32)	0.17	1.42 (0.87, 2.31)	0.16
Socioeconomic status (per 1 unit increase)	0.99 (0.94, 1.03)	0.59	1.00 (0.94, 1.06)	0.98
Child-Turcotte-Pugh category				
A	1.0 (ref)		1.0 (ref)	
B	1.13 (0.79, 1.58)	0.55	1.11 (0.78, 1.57)	0.57
C	2.69 (1.52, 4.75)	0.001	2.68 (1.52, 4.75)	0.001
Alpha-Fetoprotein (AFP) (≥10ng/ml = 1, <10ng/ml = 0)	1.14 (0.78 , 1.67)	0.49	1.16 (0.80 , 1.68)	0.43
MELD score (per 1 unit increase)	1.05 (1.02 , 1.09)	0.005	1.05 (1.01 , 1.09)	0.007
Tumor thrombus (yes = 1, no = 0)	0.95 (0.63 , 1.43)	0.80	0.96 (0.64 , 1.42)	0.82
Number of lesions				
1	1.0 (ref)		1.0 (ref)	
2-3	1.00 (0.69 , 1.45)	0.99	1.00 (0.69 , 1.45)	0.99
>3	0.99 (0.65 , 1.52)	0.98	1.00 (0.65 , 1.52)	0.98
Maximum tumor diameter (>2cm = 1, ≤2cm = 0)	1.40 (1.04 , 1.88)	0.029	1.40 (1.04 , 1.89)	0.028

© 2020 English K et al. *JAMA Network Open.*

eTable2. Univariable complete case (CC) analysis and Multiple Imputation (MI) analysis estimates for overall survival (OS) from Cox regression model: unadjusted Hazard Ratio, 95% CI and P-value.

	Complete Case Estimates (*n* = 289)		Multiple Imputation Estimates (*n* = 289, M=20)	
**Variable**	**Hazard ratio (robust 95% CIs)**	**P-value**	**Hazard ratio (robust 95% CIs)**	**P-value**
Treatment group (Time Dependent Covariate) (TACE + ablative therapy = 1, TACE alone = 0)	0.28 (0.15, 0.52)	<0.001	0.28 (0.15, 0.52)	<0.001
Age (≥65y = 1, <65y = 0)	1.30 (0.79, 2.14)	0.31	1.30 (0.79, 2.14)	0.31
Sex (female = 1, male = 0)	1.24 (0.72, 2.14)	0.44	1.24 (0.72, 2.14)	0.44
Race/Ethnicity				
Non-Hispanic	1.0 (ref)		1.0 (ref)	
White Hispanics	0.57 (0.24, 1.34)	0.20	0.57 (0.24, 1.34)	0.20
Non Hispanic Black	1.15 (0.47, 2.80)	0.77	1.14 (0.47, 2.80)	0.77
ECOG performance status				
0	1.0 (ref)		1.0 (ref)	
1	1.12 (0.62, 2.04)	0.71	1.18 (0.64, 2.18)	0.60
≥2	1.31 (0.60, 2.83)	0.50	1.38 (0.65, 2.94)	0.41
Socioeconomic status (per 1 unit increase)	1.08 (0.99, 1.18)	0.079	1.08 (0.98, 1.19)	0.082
Child-Turcotte-Pugh category				
A	1.0 (ref)		1.0 (ref)	
B	2.01 (1.21, 3.33)	0.007	2.00 (1.20, 3.32)	0.007
C	1.88 (0.49, 7.31)	0.36	1.89 (0.49, 7.33)	0.36
Alpha-Fetoprotein (AFP) (≥10ng/ml = 1, <10ng/ml = 0)	1.90 (1.08, 3.36)	0.027	1.76 (0.99, 3.11)	0.054
MELD score (per 1 unit increase)	1.02 (0.96, 1.08)	0.58	1.02 (0.96, 1.08)	0.61
Tumor thrombus (yes = 1, no = 0)	1.35 (0.81, 2.25)	0.25	1.40 (0.84, 2.32)	0.20
Number of lesions				
1	1.0 (ref)		1.0 (ref)	
2-3	1.32 (0.73, 2.39)	0.37	1.32 (0.73, 2.39)	0.37
>3	1.22 (0.65, 2.31)	0.54	1.22 (0.65, 2.31)	0.54
Maximum tumor diameter (>2cm = 1, ≤2cm = 0)	1.29 (0.76, 2.18)	0.34	1.29 (0.76, 2.17)	0.35

© 2020 English K et al. *JAMA Network Open.*

Supplementary Table E3. Multivariable complete case (CC) analysis and Multiple Imputation (MI) analysis estimates for overall survival (OS) from Cox regression model in patients with BCLC stage B or C: adjusted Hazard Ratio, robust 95% CI and P-value.

	**BCLC stage B/C Complete Case Estimates (*n* = 123 patients)**		**BCLC stage B/C Multiple Imputation Estimates (*n* = 123 patients)**	
**Variable**	**Hazard ratio (robust 95% CIs)**	**P-value**	**Hazard ratio (robust 95% CIs)**	**P-value**
Treatment group (TACE + ablative therapy = 1, TACE alone = 0)	0.33 (0.15, 0.76)	0.009	0.31 (0.14, 0.69)	0.004
Alpha-Fetoprotein (AFP) (<10ng/ml = 1, ≤10ng/ml = 0)	2.15 (0.99, 4.68)	0.054	2.08 (0.98, 4.45)	0.058
Number of lesions				
0-1	1.0 (ref)		1.0 (ref)	
2-3	2.30 (0.93, 5.76)	0.073	2.07 (0.84, 5.11)	0.12
>3	0.67 (0.29, 1.56)	0.36	0.60 (0.26, 1.34)	0.21
Socioeconomic status (per 1 unit increase)	1.20 (1.06, 1.36)	0.004	1.18 (1.04,1.33)	0.010
Maximum tumor diameter (>2cm = 1, ≤2cm = 0)	2.34 (1.01, 5.38)	0.046	2.07 (0.94, 4.55)	0.072

© 2020 English K et al. *JAMA Network Open.*
